# A Nomogram to Predict the Outcome of Fine Needle Aspiration Cytology in Head and Neck Masses

**DOI:** 10.3390/jcm8122050

**Published:** 2019-11-22

**Authors:** Ulana Kotowski, Faris F. Brkic, Oskar Koperek, Eleonore Pablik, Stefan Grasl, Matthaeus Ch. Grasl, Boban M. Erovic

**Affiliations:** 1 Department of Otorhinolaryngology and Head and Neck Surgery, Medical University of Vienna, 1090 Vienna, Austria; 2 Department of Pathology, Medical University of Vienna, 1090 Vienna, Austria; 3 Clinical Pathology Laboratory Kaserer, Koperek & Beer, 1030 Vienna, Austria; 4 CeMSIIS, Section for Medical Statistics, Medical University Vienna, 1090 Vienna, Austria; 5 Institute of Head and Neck Diseases, Evangelical Hospital Vienna, 1180 Vienna, Austria

**Keywords:** nomogram, fine needle aspiration cytology, neck lumps, salivary gland, cancer

## Abstract

Fine needle aspiration cytology (FNAC) is an important diagnostic tool for tumors of the head and neck. However, non-diagnostic or inconclusive results may occur and lead to delay in treatment. The aim of this study was to evaluate the factors that predict a successful FNAC. A retrospective search was performed to identify all patients who received an FNAC at the Department of Otorhinolaryngology, Head and Neck Surgery, Medical University of Vienna. The variables were patients’ age and sex, localization and size of the punctured structure, previous radiotherapy, experience of the head and neck surgeon, experience of the pathologist and the FNAC result. Based on these parameters, a nomogram was subsequently created to predict the probability of accurate diagnosis. After performing 1221 FNACs, the size of the punctured lesion (*p* = 0.0010), the experience of the surgeon and the pathologist (*p* = 0.00003) were important factors for a successfully procedure and reliable result. FNACs performed in nodes smaller than 20 mm had a significantly worse diagnostic outcome compared to larger nodes (*p* = 0.0004). In conclusion, the key factors for a successful FNAC are nodal size and the experience of the head and neck surgeon and the pathologist.

## 1. Introduction

The fine needle aspiration cytology (FNAC) is a well-tolerated, safe, timesaving and cost-effective diagnostic test. It is therefore widely performed in the initial evaluation of head and neck masses. As reported in literature, FNAC has a high sensitivity (72.4–100%), a high specificity (85.3–100%) as well as a high accuracy (73.3–98.0%) [1]. However, certain rates of non-diagnostic or inconclusive results have also been reported [2,3]. In case of a non-diagnostic result, the sample contains not enough cells, or it was contaminated by blood cells. Inconclusive results occur when cells have atypical characteristics but cannot be clearly assigned as malignant or benign. As an alternative an open biopsy can be performed, but a surgical intervention carries risks like bleeding, scarring, nerve injury and spillage of tumor cells [4]. In addition, some patients are not suitable for surgical procedures due to their poor general condition. Nonetheless a quick evaluation of a neck node or a parotid tumor is essential to establish the right treatment plan for the patient. Thus, FNAC is a rapid and minimal invasive diagnostic tool [5]. The accuracy of an FNAC strongly depends on the experience of the physician performing the FNAC and on the expertise of the examining pathologist [6,7].

Nomograms are well-established statistical tools to individualize risk assessment. They are a pictorial representation of a mathematical formula. Medical nomograms use clinical variables to graphically present a statistical prognostic model that predicts the probability of a clinical event such as treatment outcome of a malignant disease. Each variable is assigned a corresponding number of points. Then the cumulative score for all variables is fitted to a result scale [8].

The aim of this study was to evaluate, which parameters lead to a successful FNAC. Therefore, possible influencing factors like localization (parotid or submandibular gland, neck and thyroid) and size of the node, previous radiotherapy treatment, experience of the examiner and experience of the pathologist were evaluated. Based on these parameters, a nomogram was subsequently constructed in order to predict the probability of diagnostic FNAC outcome.

## 2. Materials and Methods

### 2.1. Patients

A database research was conducted to identify all performed FNAC procedures from 2006 to 2018 at the Department of Otorhinolaryngology, Head and Neck Surgery, Medical University of Vienna. All patients with an unclear salivary gland tumor, suspicious neck node or a thyroid node that received an FNAC for diagnostic purposes were included in this study. A suspicious node was defined as a well-palpable, painless node persisting for more than 3 months. Most patients were sent to our department by family doctors or consultants for further diagnostic workup of cervical masses. Some patients have previously undergone an imaging study. All patients underwent a careful medical history and a physical examination prior to performing FNAC. Searched variables included patients’ age and sex, previous radiation (yes/no), localization of the node, experience of the head and neck surgeon, experience of the pathologist, FNA result and histology. Node size was determined from present imaging studies (e.g., ultrasound, CT scan, MRI scan).

For the calculation of true positive/negative and false positive/negative results, FNAC results were compared with the histological result, if available.

This retrospective study was approved by the Research Ethics Board of the Medical University of Vienna (1261/2016).

### 2.2. Fine Needle Aspiration Cytology

All FNAC procedures were carried out in head and neck masses without ultrasound- guidance on well palpable and well accessible nodes. The procedure was performed at our outpatient department. As equipment, a 23 gauge needle mounted on a 20 mL syringe was used. At least two samples were taken each time and immediately after aspiration the samples were smeared out on a microscope slide. Right thereafter one slide was fixed with an alcohol-based spray fixative (M-FIX^TM^, Merck KGaA, Darmstadt, Germany) and the other one was air-dried. Subsequently, the slides were sent to the pathologist for examination. We have not observed any complications after FNAC in our cohort. The FNAC procedure was performed by different head and neck surgeons with different levels of experience (attendings and consultants). Pathologist, also with different levels of experience, evaluated the cytological smear.

The cytopathologic results were classified into four groups: a non-diagnostic group, an inconclusive group, a malignant group and a benign group.

### 2.3. Statistical Analysis

For descriptive statistics, the Statistical Package for the Social Sciences (SPSS^®^, version 24; IBM Corp., Armonk, NY, USA) was used.

For univariate and multivariate analysis, the unequivocal results were summarized as outcome “YES” and the non-diagnostic and inconclusive results were summarized as outcome “NO”. A univariate logistic regressions model was used to identify candidates for a multivariable logistic regression model. All variables which were significant at a significance level α = 0.15 were combined into a multivariable logistic regression model. Stepwise backward reduction according to the best Akaike information criterion was applied to obtain the final best multivariable logistic regression model, which also was visualized with a nomogram. As diagnostic plots showed no monotone change over the years of experience (neither for the head and neck surgeon nor for the pathologist), the experience in years was treated as categorical variable.

## 3. Results

### 3.1. Patients

Our database research revealed that 1221 FNAC biopsies were performed during a 12 year period. There were 484 female (39.6%) and 737 male (60.3%) patients. The mean patients’ age at time of FNAC was 57.4 years (SD 17 years; range 3.3–96.2 years). The median size of the punctured nodes was 25 mm (SD 14 mm; range 3–110 mm). Moreover, 11.6% of the patients had a previous radiotherapy. In total, 449 (37%) lesions were found in the parotid gland and 50 (4%) nodules were found in the submandibular gland. Furthermore, 697 (57%) cervical lymph nodes and 25 (2%) thyroid nodules were examined.

### 3.2. FNAC Results

In our cohort, 793 FNAC samples (65%) had an unequivocal result, showing clearly 519 benign and 274 malignant cells. Furthermore, 338 aspirates (27.7%) were non-diagnostic either because of lack of cells or due to blood contamination. Ninety samples (7.3%) showed atypical cells and a clear assignment to benign or malignant cells was not possible (Table 1).

Next, FNAC results were compared to the histopathological report, if available. The true positive, the true negative, the false positive and the false negative rate was 69%, 97%, 3% and 24%, respectively (Table 2).

### 3.3. Univariate Analysis

Univariate analysis showed that neither patients’ age (*p* = 0.1848) nor previous radiotherapy in the head and neck area (*p* = 0.3326) had influence on the FNAC outcome (Table 3). Furthermore, the localization (parotid or submandibular gland, cervical lymph node, thyroid) of the punctured node had no impact on the FNAC result (*p* = 0.2250). The size of the punctured node, however, had a significant influence on the outcome of an FNAC (*p* = 0.0010). Performing FNAC on a node smaller than 20 mm resulted significantly more frequently in a non-diagnostic or inconclusive result (*p* = 0.0004).

Next, we evaluated whether the experience of the head and neck surgeon and/or the pathologist had effect on FNAC outcome. Regarding the head and neck surgeon, we found no difference to the success of FNAC over the years of training (*p* = 0.2341). The worst performance was after five years of practicing FNAC (*p* = 0.0843) compared to the first year of training. Since the total number of performed FNAC differed between each colleague over the years, we also summarized the experience of the head and neck surgeon in “beginner”, “intermediate” and “expert”. Previous studies have shown that more than 50 FNACs are necessary to gain sufficient expertise [7,9]. In our study the beginner had performed up to 50 FNAC procedures; the intermediate up to 100 and the expert had performed over 100 FNAC procedures. In an overall analysis, our data shows no significant difference between beginner, intermediate and expert training level (*p* = 0.1110). However, the intermediate experienced head and neck surgeon had more often non-diagnostic or inconclusive results compared to the beginner (*p* = 0.0364).

The experience of the pathologist in years had a much greater influence on the success of an FNAC (*p* = 0.00003). The worst performance was found after three and four years of training (*p* = 0.0058 and *p* = 0.0057). We also stratified the pathologist into “beginner”, “intermediate” and “expert” groups. However, there was no statistically significant difference between those groups (*p* = 0.2715).

### 3.4. Multivariate Analysis

The multivariate analysis showed that the size of the punctured node and the experience of the pathologist in years had a significant impact to predict the probability of FNAC outcome (Table 4).

### 3.5. Nomogram

**Figure 1:**
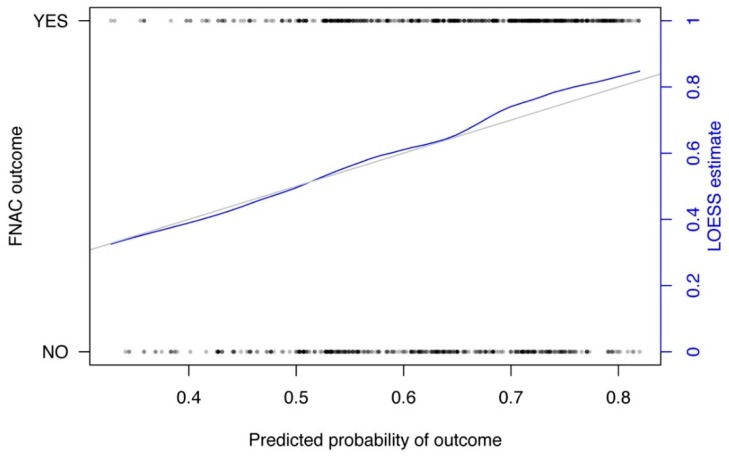
Calibration curve of the nomogram. The *X*-axis represents the predicted probability of diagnosis whereas FNAC outcome is shown on the *Y*-axis. “YES” are unequivocal results (e.g., clear benign or malignant cells). “NO” are non-diagnostic and inconclusive results. The diagonal line represents the ideal performance and the curved line represents the performance of the nomogram.

**Figure 2:**
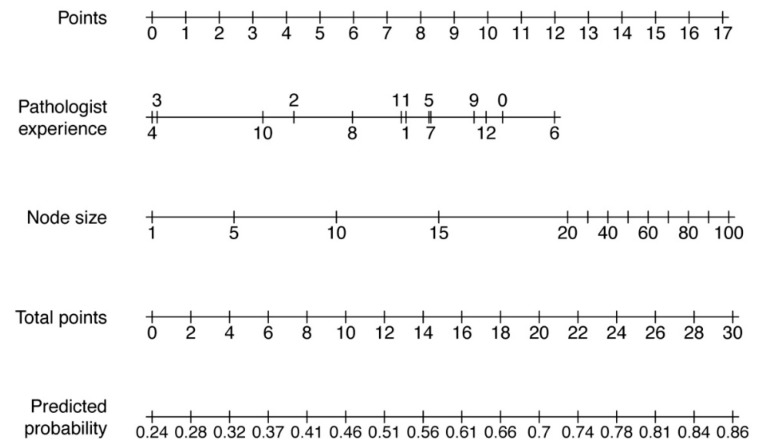
Nomogram to predict the outcome of an FNAC. The points for each variable (pathologist experience in years and node size in mm) are determined by drawing a line upwards to the first axis. After counting all points, a line is drawn down from the axis with the total points to the axis of predicted probability to determine the probability of accurate FNAC.

**Figure 3:**
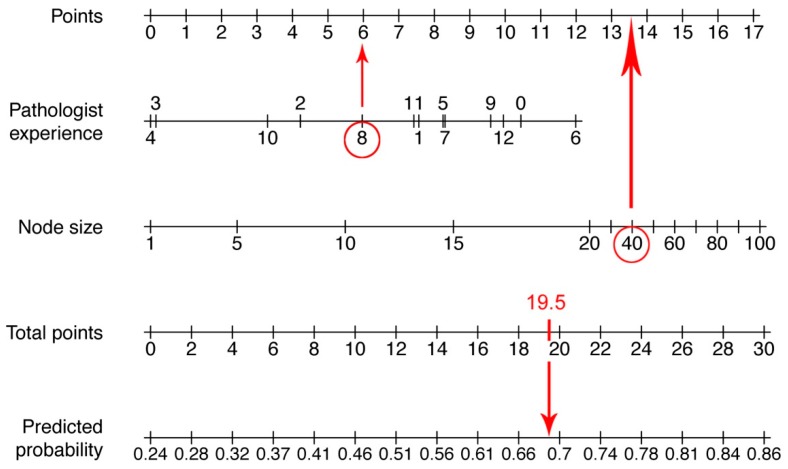
An example of how to use a nomogram. An FNAC was performed in a 40 mm node (13.5 points) and examined by a pathologist with 8 years of experience (6 points). The total points are 19.5 and the predicted probability is therefore approximately 0.7 (70%).

To predict the FNAC outcome based on the parameters size of the punctured node and the experience of the pathologist in years, a nomogram was calculated. The calibration curve and the nomogram are depicted in Figure 1 and Figure 2. The pathologist experience and the node size are located on different axes. The points for each variable are determined by drawing a line upwards to the points-axis. Then all points are summed up. The total points are located on a separate axis. A line is drawn down to the axis of predicted probability to determine the probability of reliable FNAC. An example of how to work with a nomogram is shown in Figure 3.

## 4. Discussion

Palpable cervical masses can arise from a variety of pathologic conditions such as inflammatory diseases and neoplastic processes. For rapid diagnostic workup, FNAC is a well-established procedure that can be performed in the outpatient setting with minimal to no risk of complications [7,8]. The predictive value of a positive FNAC is high but there is a certain rate of non-diagnostic results [10]. In this work, various factors were examined which could influence the success of an FNAC. Based on these variables, a nomogram was calculated to predict the probability of an accurate diagnosis.

Our study evaluated 1221 FNAC results. Both, univariate and multivariate analysis showed that the size of the node is the deciding factor for a successful FNAC performed without ultrasound guidance. The probability of obtaining a usable result was significantly lower in nodes smaller than 20 mm. Literature research revealed that not many studies address to the question whether the size of a punctured node has an impact on the success of an FNAC. One study about ultrasound-guided FNAC performed in thyroid nodules reported that the non-diagnostic rate was higher in nodules smaller than 1 cm [7]. Another study found that ultrasound-guided FNAC in thyroid nodules smaller than 5 mm in maximum diameter was less successful [11]. In contrast, a study in salivary glands found no impact of the lesion size on FNAC outcome [12].

An additional important factor for the success of FNAC in our cohort was the experience of the pathologist. The evaluation of a cytologic sample can be challenging for a pathologist, therefore, these specimens should be assessed by a well-trained cytopathologist [13]. In the literature, it is repeatedly emphasized that the experience of the pathologist is crucial for the success of an FNAC [1]; however, we did not find any information about when a pathologist is considered as experienced. A study in thyroid nodules postulated that a pathologist requires at least 50 FNACs to gain sufficient expertise [8]. Interestingly, our analysis shows that the results of pathologists were least reliable after three and four years of training. An explanation can be given by the fact that after three and four years of training, the young pathologist are evaluating the cytopathological slides by themselves and are less likely to counsel an experienced colleague. Lacking many years of experience, this may lead to more inconclusive results. However, when looking at the experience level in terms of “beginner”, “intermediate” and “expert” there was no difference in obtaining unequivocal results. 

Regarding the experience of the head and neck surgeon, univariate analysis showed that surgeon at intermediate training levels had worse FNAC results compared to beginners. An explanation for this phenomenon could be that, similar to pathologist, physicians with intermediate experience are not under supervision anymore but yet do not have sufficient expertise. Therefore we suggest that in doubt, colleagues with intermediate expertise level should more often consult more experienced colleagues. Other studies have also shown that the accuracy rate improves with the experience of the operator [14]. However, a study that performed 193 FNACs in head and neck masses, found no differences in the results of FNACs performed by an attending or a resident [15].

Multivariate analysis showed that beside the node size, only the experience of the pathologist in years has a significant influence on the success of an FNAC. Taken together, the experience of the pathologist has much greater influence on the FNAC result than the experience of the surgeon.

In our cohort 65% FNAC samples obtained an unequivocal result, in 7.3% atypical cells were found and a clear assignment to benign or malignant cells was not possible. In 27.7% FNAC specimens were non-diagnostic due to lack of cells or blood contamination. Our results are comparable to the literature. A study about surgeon-performed ultrasound guided FNAC in thyroid nodules reported a non-diagnostic rate of 23%. Nodules with predominant cystic component and resident-performed FNAC were significantly associated with non-diagnostic cytology [7]. As described in literature, a further factor that can improve the outcome of FNAC is the onsite assessment. A cytopathologist that immediately evaluates the cytologic smear can easily detect inadequate samples [16,17]. Furthermore, the ultrasound guided technique is a useful tool to improve the diagnostic outcome of FNAC [18,19]. Possible limitations of FNAC are that the lesion may be difficult to access due to the localization and that some malignancies, such as lymphomas are difficult to be diagnosed by FNAC [1]. As an alternative an ultrasound-guided core biopsy can be performed [20].

In our cohort, patients with equivocal FNAC results received either an ultrasound follow-up, further imaging studies (CT or MRI scan) or an open biopsy, depending on the suspected diagnosis. To improve the assessment of head and neck masses, our department recently introduced the utility of an ultrasound-guided FNAC and/or core biopsy.

Nomograms are statistical tools to predict the probability of outcome and to individualize risk assessment. Based on different criteria an objective and personalized decision without individual bias of the physician can be made. In clinical practice, they are often used to predict prognosis and treatment outcomes of malignant diseases [21]. In particular, nomograms for stratifying the risk of malignancy in thyroid nodules were established recently by different study groups [22,23].

We have developed a nomogram to predict the diagnostic outcome of FNAC, as non-diagnostic or inconclusive results delay diagnosis and possible treatment. Multivariate analysis showed that among all factors that have a potential effect on the success of an FNAC, only the size of the node and the experience of the pathologist in years have a statistically significant impact on FNAC outcome. With the aid of this diagnostic tool, it will be now less challenging to decide whether to perform an FNAC or to consider another diagnostic method. A limitation of this study is that an external validation of our nomogram has not been done. However, this is the first attempt to develop an instrument to predict the individual success of an FNAC.

## 5. Conclusions

In conclusion, the success of an FNAC depends on the size of the punctured lesion and on the experience of the pathologist and the head and neck surgeon. A newly established nomogram will facilitate in future the decision whether to perform an FNAC in a particular patient or if other diagnostic methods should be considered.

## Figures and Tables

**Table 1:** Total fine needle aspiration cytology (FNAC) results.

	Benign Lesion	Malignant Lesion	No Histology Available	Total Number
**Non-diagnostic FNAC**	124 (10.2%)	97 (7.9%)	117 (9.6%)	338 (27.7%)
**Inconclusive FNAC**	23 (1.8%)	51 (4.2%)	16 (1.3%)	90 (7.3%)
**Benign FNAC**	273 (22.4%)	93 (7.6%)	153 (12.5%)	519 (42.5%)
**Malignant FNAC**	8 (0.7%)	204 (16.7%)	62 (5.1%)	274 (22.5%)
**Total Number**	428 (35.1%)	445 (36.4%)	348 (28.5%)	1221 (100%)

**Table 2:** True negative, false negative, false positive and true positive FNAC in case of available histology.

	Benign Lesion	Malignant Lesion
**Benign FNAC**	True Negative 273 (97%)	False Negative 93 (31%)
**Malignant FNAC**	False Positive 8 (3%)	True Positive 204 (69%)

**Table 3:** *P*-values of univariate model.

Variables	*p*-Value
Patient‘s age	0.1848
Node size (metric scale)	0.0010
Node > 20 mm (yes/no)	0.0004
Previous radiotherapy	0.3326
Localization of node	0.2250
Pathologist experience in years	0.00003
Pathologist experience in levels	0.2715
Head and neck surgeon experience in years	0.2341
Head and neck surgeon experience in levels	0.1110

**Table 4:** *P*-values for final logistic regression model (multivariate analysis).

Variables	*p*-Value
Pathologist experience after 1 year	0.4498
Pathologist experience after 2 years	0.1025
Pathologist experience after 3 years	0.0079
Pathologist experience after 4 years	0.0080
Pathologist experience after 5 years	0.5951
Pathologist experience after 6 years	0.7170
Pathologist experience after 7 years	0.6074
Pathologist experience after 8 years	0.3104
Pathologist experience after 9 years	0.8464
Pathologist experience after 10 years	0.1008
Pathologist experience after 11 years	0.6699
Pathologist experience after 12 years	0.9483
Node size (metric scale)	0.0109
Node > 20 mm (yes/no)	0.0311
